# Design of effective self-powered SnS_2_/halide perovskite photo-detection system based on triboelectric nanogenerator by regarding circuit impedance

**DOI:** 10.1038/s41598-022-11327-0

**Published:** 2022-05-04

**Authors:** Leyla Shooshtari, Soheil Ghods, Raheleh Mohammadpour, Ali Esfandiar, Azam Iraji zad

**Affiliations:** 1 Institute for Nanoscience and Nanotechnology, Sharif University of Technology, Tehran, 14588-89694 Iran; 2 Physics Department, Sharif University of Technology, Tehran, 11365-9161 Iran

**Keywords:** Energy science and technology, Optics and photonics, Physics

## Abstract

Self-powered detectors based on triboelectric nanogenerators (TENG) have been considered because of their capability to convert ambient mechanical energy to electrical out-put signal, instead of conventional usage of electrochemical batteries as power sources. In this regard, the self-powered photodetectors have been designed through totally two lay out called passive and active circuit. in former model, impedance matching between the TENG and the resistance of the circuit’s elements is crucial, which is not investigated systematically till now. In this paper, a cost effective novel planar photodetector (PD) based on heterojunction of SnS_2_ sheets and Cs_0.05_(FA_0.83_ MA_0.17_)_0.95_Pb(I_0.83_Br_0.17_)_3_ three cationic lead iodide based perovskite (PVK) layer fabricated which powered by graphene oxide (GO) paper and Kapton based contact-separated TENG (CS-TENG). To achieve the high performance of this device, the proper range of the load resistances in the circuit regards to TENG’s characterization has been studied. In the next steps, the integrated self-powered photo-detection system was designed by applying Kapton/FTO and hand/FTO TENG, separately, in the proposed impedance matching circuit. The calculated D* of integrated self-powered SnS_2_/PVK supplied by tapping the Kapton and hand on FTO is 2.83 × 10^10^ and 1.10 × 10^13^ Jones under the 10 mW/cm^2^ of white light intensity, the investigations determine that for designing significate performance of self-powered PD supplied by TENG, the existence of the load resistance with the well match amount to the utilized TENG is crucial. Our results which can be generalized to other types of passive self-powered sensors, are substantial to both academia and industry concepts.

## Introduction

On account of the improvement the Internet of things (IoTs) and smart devices, our lives have been noticeably facilitated in the past few years. Machines and devices are becoming more ingenious with the help of artificial intelligence and various sensors^1,2^. So, integrated circuits are necessary to provide convenient and effectual communication^3^ Since the first report on TENG by Wang’s group in 2012^4^, triboelectric systems have been recognized as a proper choice to harvest and convert the energy from the environment^5,6^. Photodetectors, as one of the most significant types of sensors that can precisely convert incident light into electrical signals have attracted increasing attention in recent years. Various applications including photo-sensors, spectral analysis^7,8^, environment monitoring^9^, communication devices^10^, imaging^11^, take advantage of narrow band or broad band photodetectors from ultraviolet to terahertz wavelenght. Literature reviews show that the heterojunction/heterostructure based on 2D/3D materials have been widely used in PD applications. In fact, to attain high performance of PDs based heterojunction, the built-in electrical field is needed to suppress the photogenerated recombination and stimulating collection^12^. Although, Si based PDs offer reliably high performance results, their complexity and expensive manufacturing process have limited their expansion and adoptability for industrial purposes^13–15^. Hence, most available PDs are designed based on external power supplies such as electrochemical batteries for signal production and processing, their design not only increases the sensor’s dimension and weight, but also creates limitations for sensor maintenances^16^ which is not proper in the IoTs. In 2014, ZH Lin et al. and Zheng et al. represented an investigation on the self-powered PD based on TENG system^3,17^, and since then, self-driven PDs have been extensively investigated^2,5,9,18–20^. These devices can find potential applications in health monitoring systems such as heart checking^21^ and health protection from some detrimental radiation such as high levels of UV radiance^22^.

But in the other hand, even though TENGs could be promise for using in wearable electronics, they still inevitably have limitations in power generation, sensing range, sensitivity, and also the sensing domain for the intrinsic limitations of electrification^23–25^. Moreover, due to high voltage, low current, and alternating current output of the TENGs, they cannot be used in order to supply power to electronic devices effectively without using power management circuits (PMCs) based on the LC modules. There are several reports that describe the importance of the impedance matching of the TENG and PMC units for better energy storage efficiency of the pulsed-TENG^26,27^. Without using the PMC unit, there are some challenges as a result of synching the TENG, as the power supply, and the consumption element such as the PD device. These challenges include the process of matching the resistance of the device and the impedance of the TENG to achieve effective performance of the self-powered system^6,28^.

In this study an efficient battery-free photodetector based on bulk heterojunction SnS_2_ nanosheets and perovskite materials has been designed and powered employing three different TENGs (GO paper/ Kapton, FTO/Kapton and hand/ FTO). In the first step for circuit designing to have better performance of the photodetector in coupling with TENG, the effect load resistance amount in the circuit on the impedance matching the TENG and the inner resistance of the photodetector, has been investigated through output current amplitude. The investigation, shows that to achieve the high amount of the photocurrent, the load resistance should be positioned in both critical zone of the out-put voltage of the TENG and the resistance range of high power density production of the TENG. In the second step, for investigation the effect of the dark resistance of the photodetector on out-put current of the self-powered photodetector, a device with very lower initial resistance (All-oxide Cu_2_O/ZnO photodetector) has been used with and without different load resistance in the circuit; in this regard, it is concluding that the initial resistance is too important to have proper design impedance matching circuit.

In the next step, an integrated self-powered SnS_2_/PVK photodetector by tapping the Kapton and hand on the FTO through impedance matching has been regarded through using proper load resistance. It is determined that, by tapping the hand on the FTO, the responsivity and detectivity of the self-powered SnS_2_/PVK enhance to 1.10 × 10^13^ Jones in compare with self-powered photodetection system by regarding the GO paper/Kapton TENG with detectivity of 2.56 × 10^10^ Jones. The results obtained in this research can be very significant in future designs of self-powered sensors.

## Results and discussion

### Photodetector characterization

**Figure 1:**
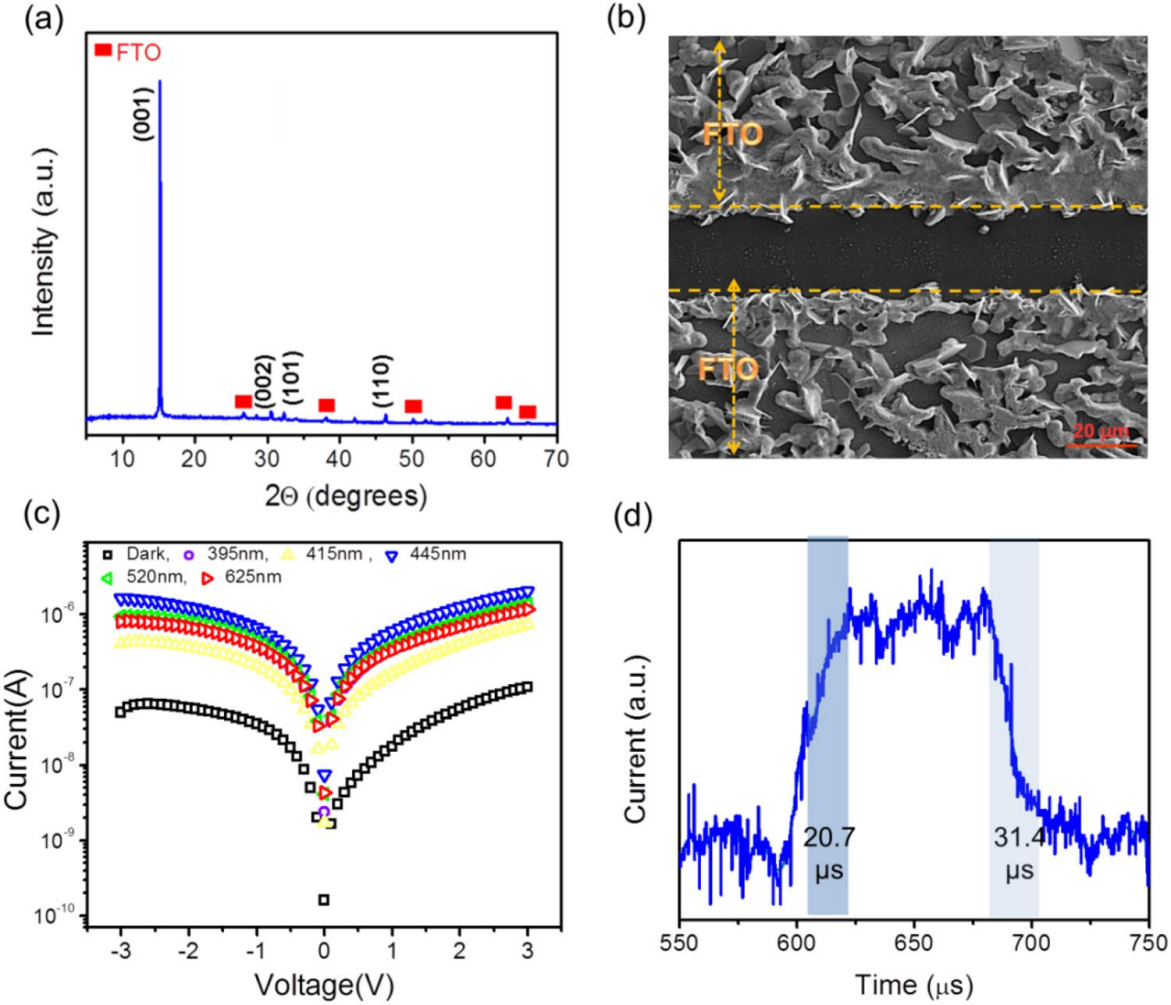
(**a**) SnS_2_/PVK based PD characterization. The XRD pattern of the vertically grown SnS_2_ nanosheets on the FTO. (**b**) Top view FESEM analysis of the SnS_2_/PVK, homogenous covering of PVK layer occurs on the SnS_2_ nanosheet films. (**c**) The semi-log current of SnS_2_/PVK photodetector vs. applying voltage for different wavelengths of 395 nm, 415 nm, 445 nm, 520 nm, and 635 nm with the same intensity of 5 mW/cm^2^. (**d**) the rising and falling time of the SnS_2_/PVK under a wavelength of 445 nm with the intensity of 5 mW/cm^2^.

The XRD patterns of pristine SnS_2_, which are in good agreement with the reference card of JCPDS no 01-83-1705, show the preferred orientation of (001) facets for the grown SnS_2_ nanosheets (Fig. 1a), confirming vertical growth with semi hexagonal nanosheets as it is observed by optical imaging (Fig. S1a). Vertical growth is due to the surface diffusion of incoming species and lattice mismatched at the initial stage of growth and later in the (001) preferred orientation^29,30^.

Moreover, FESEM analysis of the vertically grown SnS_2_ on patterned FTO shows the semi hexagonal SnS_2_ nanosheets which is grown on the FTO/glass substrate create electrical bridges over the patterned FTO and lead to the cost effective fabrication of the planar photodetector (Fig. S1b). In addition, the top view FESEM analysis of SnS_2_/PVK displays a homogenous covering of PVK on the SnS_2_ nanosheets which is promising structure for better charge creation and separation in heterojunction based photodetector (Fig. 1b). The XRD of heterojunction of SnS_2_/ PVK has been displayed in Fig. S1c. Obviously the crystal structure of the SnS2 nanosheet with the prefer orientation (001) has been preserved and the phase crystal of the pure perovskite has been formed on the SnS2 layer, as well. The XRD pattern of the PVK (Cs_0.05_(FA_0.83_MA_0.17_)_0.95_Pb(I_0.83_Br_0.17_)_3_) layer shows that crystalline planes of Cs_0.05_ M perovskite has been created (Fig. S1d) which is in accordance with the other reports^31^.

The *I–V* characterization of the SnS_2_/PVK photodetector was performed in the dark and under different light illumination with different wavelengths of 395 nm, 415 nm, 445 nm, 500 nm, and 625 nm with the same 5 mW/cm^2^ intensity, at the bias voltage range of 3 V to − 3 V. It was observed that the proposed photodetector was greatly sensitive to the visible light illumination particularly to the 445 nm wavelength. The symmetric behavior of the *I–V* curve is due to the formation of equal electrical contacts of FTO in the facile proposed planar photodetector structure (Fig. S2a). Regarding the symmetric *I–V* curve behavior of the SnS_2_/PVK photodetector and based on the semi- log plot of the current versus voltage (Fig. 1c), the equivalent resistance of this photodetector in the dark (R_SnS2/PVK-dark_) is about 45 MΩ, while R_SnS2/PVK-illuminated_ reduced to the order of 2 MΩ under visible wavelengths. Moreover, under the exposure of the light with 445 nm wavelength, the device not only shows a stable behavior under light on–off switching for a long period (Fig. S2b), but also it exhibits an outstanding rising and falling time to the intensity (the time between 10 and 90% of the maximum photocurrent) of 20.7 µs and 31.6 µs, respectively (Fig. 1d). Therefore, this device is a suitable candidate for the contact –separated mode triboelectric nanogenerators even with high frequency motion. More characteristic of the SnS_2_/PVK photodetector can be found here^32^.

For evaluating the resistance of the photodetector and its effect in the impedance circuit through coupling with TENG, another device based on ZnO/Cu_2_O heterojunction has been fabricated. the XRD characterization of electrodeposited ZnO and Cu_2_O layers on the transparent conductive oxide as the substrate shows the (100), (002), (101), (102) and (110) peaks for ZnO layer and (110), (111), (220) and (222) for Cu_2_O layer (Figs. S3a,b), which are in accordance with the other reports, confirm the proper formation of these materials^33,34^. Moreover, the FESEM analysis of the ZnO deposition shows the homogenous and good coverage of the ZnO nanoparticulated films on the substrate (Fig. S3c). The pyramided morphology of cuprous oxide layer (Fig. S3d) is consistent with other studies in the literature^35^.

The cross-section FESEM analysis of the FTO/ZnO/Cu_2_O/Au structure (Fig. S3e) confirms a well stacking of ZnO/Cu_2_O layers without any pinhole formed through the electrodeposition process. Figure S3f shows the *I–V* curves of the device without the presence of light and under light illumination, determining 34 kΩ and 7 kΩ of resistance for the device in the dark and under incident white LED illumination, respectively. So the resistance of all oxide photodetector is 300 and 1000 times lower than the SnS_2_/PVK photodetector in the dark and under light respectively. so, if the inner resistance will be a critical point, should be effective in the impedance matching circuit, as mentioned later.

### Triboelectric nanogenerator characterization

**Figure 2:**
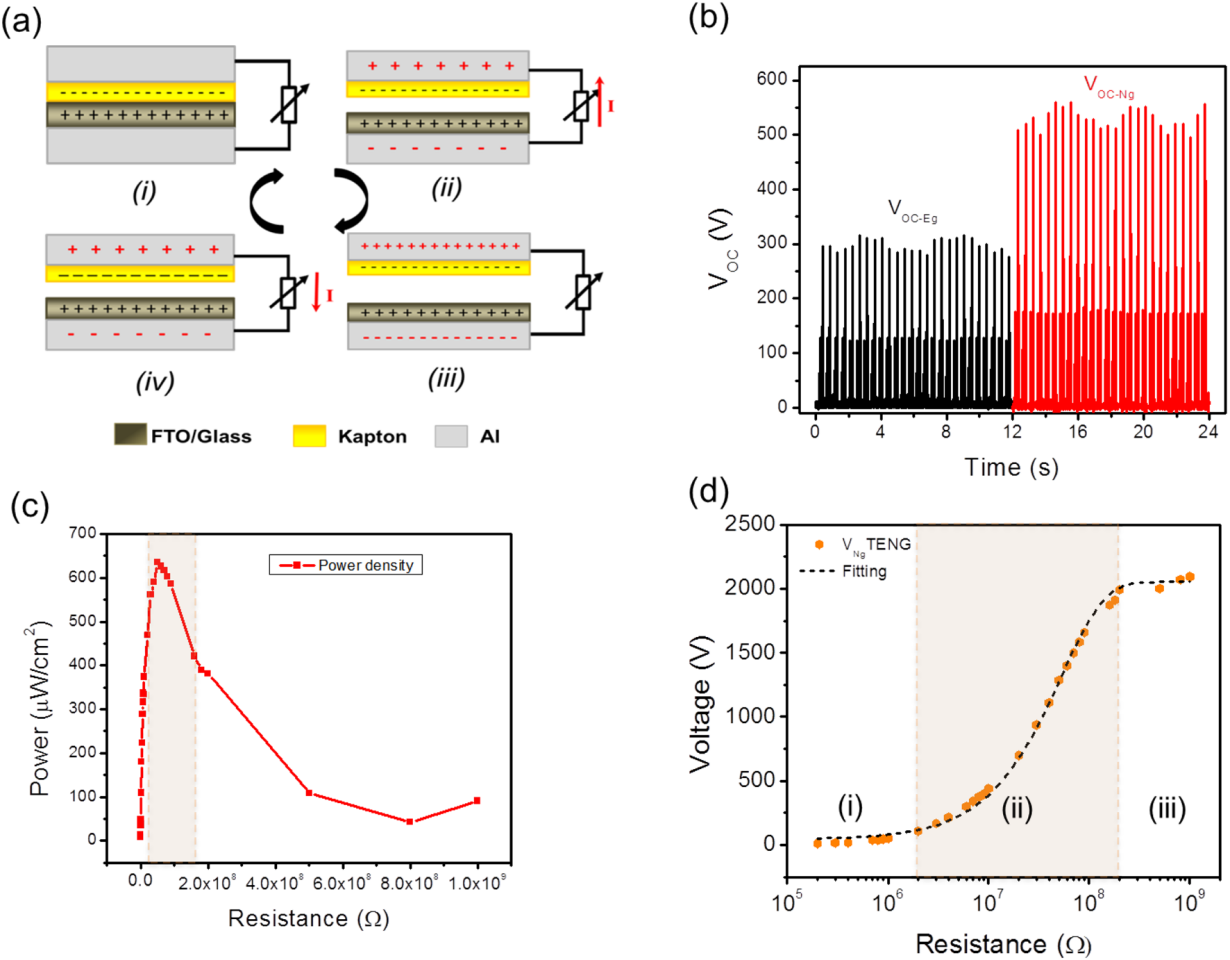
(**a**) The GO/Kapton based CS- TENG properties. The schematic representation of the GO paper/Kapton TENG mechanism (**b**) The rectified output voltage of TENG progress vs. time in the grounded and non-grounded systems. (**c**) The evolution of the power density versus load resistance (R_L_). (**d**) The output voltage of the TENG over time; three zones of very low (i), medium (ii) and large enough (iii) which is more clear in the semi-log of the TENG current vs. R_L_.

The CS-TENG based on GO paper and Kapton was employed to drive the PD based on impedance matching model circuit. The process of power generation of the TENG is established on the coupling effect of tribolectrification and electrostatic induction^36–38^. As shown briefly in Fig. 2a, when external force is in pressing mode, it brings the Kapton and GO layers in contact with each other creating triboelectric charges at two interfaces (step i); by releasing the external force, the Kapton layer moves upward and separates from the charged GO-paper triboelectric layers. The two aluminum layers as the contact surfaces reaping the induced charges due to the induction effect. So an electrical potential difference is created which drives free electrons from the back electrode to the top electrode and thus a current pulse is generated in the external circuit (step ii). After the Kapton layer moves upward in its maximum state, the charges reach the balanced state so the current flows in the external device are stopped (step iii); opposing the pressing process, free charges flow back in the circuit which complete the cycle of electricity generation (step iv)^39–41^. The previous study on the GO paper/Kapton based TENG clarified its feasibility to produce on a large scale with remarkable stability over time^42^.

To characterize the output performance of the TENG, measuring current and output voltage versus the external load resistance is necessary. Normally, this measurement is performed by using single voltmeter, so one electrode of the TENG is grounded, and the charges on this electrode should be conveyed to the ground (Fig. S4a) which causes mistakes in measuring the actual voltage values of the TENG. In this regards, W. Zhang et al. developed a non-grounded method by disconnecting both electrodes of the TENG from the ground^43^. Based on this comprehensive study, to measure the V_OC_ of the TENG, as shown in Fig. S4b, two voltmeters are connected in series with their ground terminals connects to each other and the other terminals are connected to the TENG. Figure S4c shows the graph of transient V_OC_ for CS-GO paper/Kapton in grounded and non-grounded modes. It’s clear that the V_OC-Ng_ is about 530 V while the V_OC-Eg_ is about 340 V, which is about less than twice as much.

To enhance the performance of the TENG as the external power supply for the considered PD, a rectifier bridge was used in the grounded and non-grounded circuits as shown in Fig. S4d (i) and (ii), respectively. Figure 2b shows the rectified output voltage which keeps the highest amplitude of non-rectified voltage (500 V), as rectifier bridge’s inner impedance is not very high^44^. In this study, to characterize the TENG’s parameters with regard to load resistance, an ammeter was placed in series connection with two similar resistors to accomplish the non-grounded configuration (Fig. S4e). Consequently, the electrode of the TENG was disconnected from the ground because the ground terminal of the ammeter was connected to another resistor^43^. In addition, Fig. S4f and Fig. 2c show the behavior of the non-grounded current and power density of the GO paper/Kapton TENG versus different load resistance, respectively. The maximum output current and power density values are approximately 60 µA and 6.35 mW/cm^2^. Hence, the inherent capacitance impedance of the TENG is mainly determined by total impedance of the circuit and the output voltage is directly related to the load resistance. For better illustration, the non-grounded output voltage is traced in the semi-logarithmic scale vs. load resistance (Fig. 2d). So, three regions of low (i), medium (ii) and large (iii) voltage ranges become observable^45–47^. The maximum output voltage of the Go paper/Kapton based TENG is about 2000 V, which is higher the voltage value measured by voltmeters. This phenomenon which reported before through the grounded circuit measurements^48,49^, should be come from the internal capacitance of the voltmeter^50^ which is completely described by W. Zhang et al.^51^ The resistance variation which creates the high power density range (i.e. covering 2/3 of the maximum power density output) is in the assortment of 10 MΩ < R_P Max-GO/Kapton_ < 160 MΩ, while the load resistance of 20 MΩ < R_V-Ng-GO/Kapton_ < 200 MΩ, leads to form the active zone of output voltage (i.e. region ii) of GO paper/Kapton TENG. These regions have been illustrated as colored windows in the Fig. 2c,d

### The self-powered SnS_2_/PVK PD based on GO/kapton TENG characterization

The circuit for supplying the PD by TENG as the power supply is shown in Fig. S5a. It contains a rectifying bridge, ammeter and the load resistance. In general, many rectifiers will be needed for parallel connecting of alternating current (AC)-TENGs to increase the harvested power since they are not necessarily synchronized, which causes inconvenience in practical use^52^. So as the output voltage of (AC) CS-TENG is an signal with high voltage peaks, it cannot be directly used to drive the photodetector^28^ Therefore, a rectifier is needed to transform the AC signal to a direct current (DC) signal and based on this information we tried to use this element in our impedance matching circuit. By comparing the Figs. S4e and S5a, by the ampere meter between the load resistance and the SnS_2_/PVK device, the non-grounded design has been preserved, even by using the rectifier element in the circuit.

**Figure 3:**
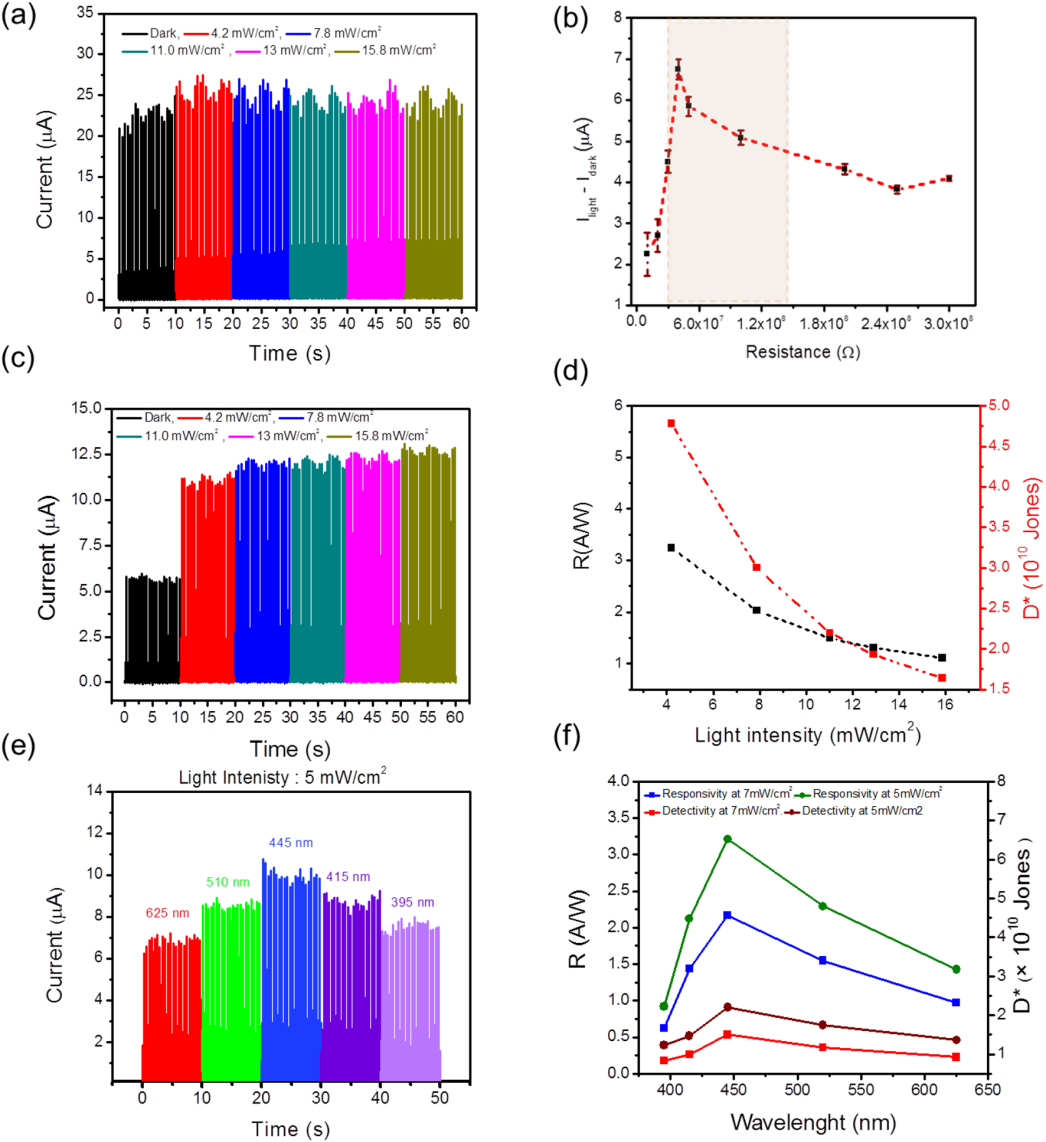
The properties of self-powered SnS_2_/PVK by GO/Kapton CS-TENG in different situations. The evolution of (**a**) current amplitude of vs. time under the different intensities of 445 nm wavelength of incident light without using any load resistance in the circuit (**b**) The *I*_*light*_*–I*_*dark*_ diversity vs. different load resistances under the illumination of 445 nm wavelength with 11 mW/cm^2^. (**c**) The current amplitude and (**d**) the subsequent calculated R and D* parameter of the self-powered SnS_2_/PVK supplied by GO paper/kapton TENG by using the 50 MΩ of load resistance in the circuit under different intensity of 445 nm wavelength. (**e**) The current amplitude and (**d**) the subsequent R and D* parameter of the self-powered SnS_2_/PVK supplied by GO paper/kapton TENG .

Since the variation between the dark resistance and the light resistance of SnS_2_/PVK structure is about 40%, it is expected that the PD in the proposed self-powered circuit shows an impressive detection of the light. But astonishingly, as Fig. 3a shows, the difference between current amplitude in the dark and under different intensities of light without using any load resistance is negligible. When the PD is exposed to the illumination, R_SnS2/PVK-illuminated_ comes to 2 MΩ. This resistance, which is in series with the GO paper/TENG in the proposed circuit, is neither in the range of effective power density nor did the active zone of this TENG (highlight part of Fig. 2c,d, respectively). So, not a noticeable diversity of the current amplitude in the dark and under different light illumination has been observable. Therefore, it is predicted that by adding the load resistance in the circuit and relocating the resistance in the range of both power density and the active zone of GO paper/Kapton TENG, the difference between dark and light current become perceptible. In this regard, different load resistance in the circuit has been used to evaluate the performance of the SnS_2_/PVK PD; As it is clear in highlighted part of Fig. 3b by changing the load resistance in the range of 30 MΩ < R _ΔI–GO/Kapton_ < 150 MΩ, the *I*_*light*_*–I*_*dark*_ shows the maximum range of variation. Obviously, this range of the resistance is the subset of the common resistance range of high power density and output voltage of GO paper/Kapton TENG, which is about 20 MΩ < R _COMN-GO/Kapton_ < 160 MΩ.

As it’s predicated, by using the load resistance in the range of R_COMN-GO/Kapton_, the current amplitude of SnS_2_/PVK PD has a remarkable change in the dark and under illumination. Figure 3c, shows the current amplitude of the self-powered SnS_2_/PVK under different light intensities of 445 nm wavelength illumination different intensities, by using the optimal load resistance of the 50 MΩ. On account of the ohm’s law, the inner resistance of the device is affected by photoelectric effect which shows the relation of the inner resistance by the light illumination intenistiy^53^. Hence, the expression of current behavior for the proposed circuit (Fig. S5b) is obtained as follows:

1 $$ I = \frac{{V_{{OC{ }\left( {TENG} \right)}} }}{{R_{i} + R_{L} }} $$

where R_i_ is the inner resistance of the PD and R_L_ is the load resistance in the circuit. To evaluate the PD’s performance responsivity of PD which is the criterion of the generated photocurrent per unit of the light intensity in the PD device can be calculated by the following equation^54^:

2 $$ R = \frac{\Delta I}{{P \cdot S}} $$

The detectivity D* of the PD is a figure of merit, defined as the inverse of the noise-equivalent power (NEP). The larger the detectivity of a photodetector, the more appropriateness of detecting weak signals which compete with the detector’s noise. This parameter can be achieved through the following equation^55^:

3 $$ D^{*} = R\sqrt {\frac{S}{{(2eI_{d} )}}} $$

where in these two equations, *ΔI* is the difference between light and dark current and *P* is the light power, *S* is the effective illuminated area; $e$ stands for the elementary charge and _Id_ is the dark current.

Figure 3d displays the evolution of these parameters versus different light intensities of 445 nm wavelength by using the best optimized load resistance of 50 MΩ. The best record of R and D* is about 3.24 A/W and 4.78 × 10^10^ Jones. As the prepared SnS_2_/PVK PD is a broadband visible wavelength detector, other sources with the wavelengths of 395 nm, 415 nm, 520 nm, and 625 nm were selected, while the R_L_ = 50 MΩ keeps the TENG as energy supplier in the high amount of power density and active region of performance. As expected, the current amplitude of the SnS_2_/PVK PD under different wavelengths and intensities of 5 mW/cm^2^ and 7.5 mW/cm^2^ display the highest output under illumination of 445 nm wavelength as shown in Fig. 3e and Fig. S5c, respectively. The normalized current variation of the self-powered PD under different wavelengths with the intensities of 5 mW/cm^2^ and 7.5 mW/cm^2^ is very similar to the trend of current evolution of SnS_2_/PVK vs. light illumination wavelengths when supplied by the battery (Fig. S5d). Calculating the PD’s parameters through different wavelengths displays the greatest values of 2.26 A/W and 3.23 A/W of responsivity and 1.95 × 10^10^ Jones and 2.83 × 10^10^ Jones for detectivity under illumination of 445 nm, with the intensities of 5 and 7.5 mW/cm^2^, respectively (Fig. 3f).

The final step to complete the investigation of device design optimization, is evaluating the effect of inner resistance of the device. As mentioned before the ZnO/Cu_2_O device has very low resistance about KΩ in the dark and under light illumination. By putting this device in the circuit including the GO papar/Kapton TENG, no current detection happened in the dark and under illumination. Even by adding several load resistances in the proposed impedance matching circuit, photocurrent shows no enhancement in compare to the dark current of the ZnO/Cu_2_O photodetector (Table S1). So, to achieve the efficient self-powered photodetector coupled with TENG, not only finding proper load resistance is important, but also the dark resistance of the device should be in the range of both density power and the critical zone of the out-put voltage of the TENG. Hence, as the SnS_2_/PVK PD obeys this critical situation, this self-powered photodetector can detect the different illumination intensity and its performance optimized by using proper load resistance in the impedance matching circuit.

### Integrated self-powered PD based on TENG

**Figure 4:**
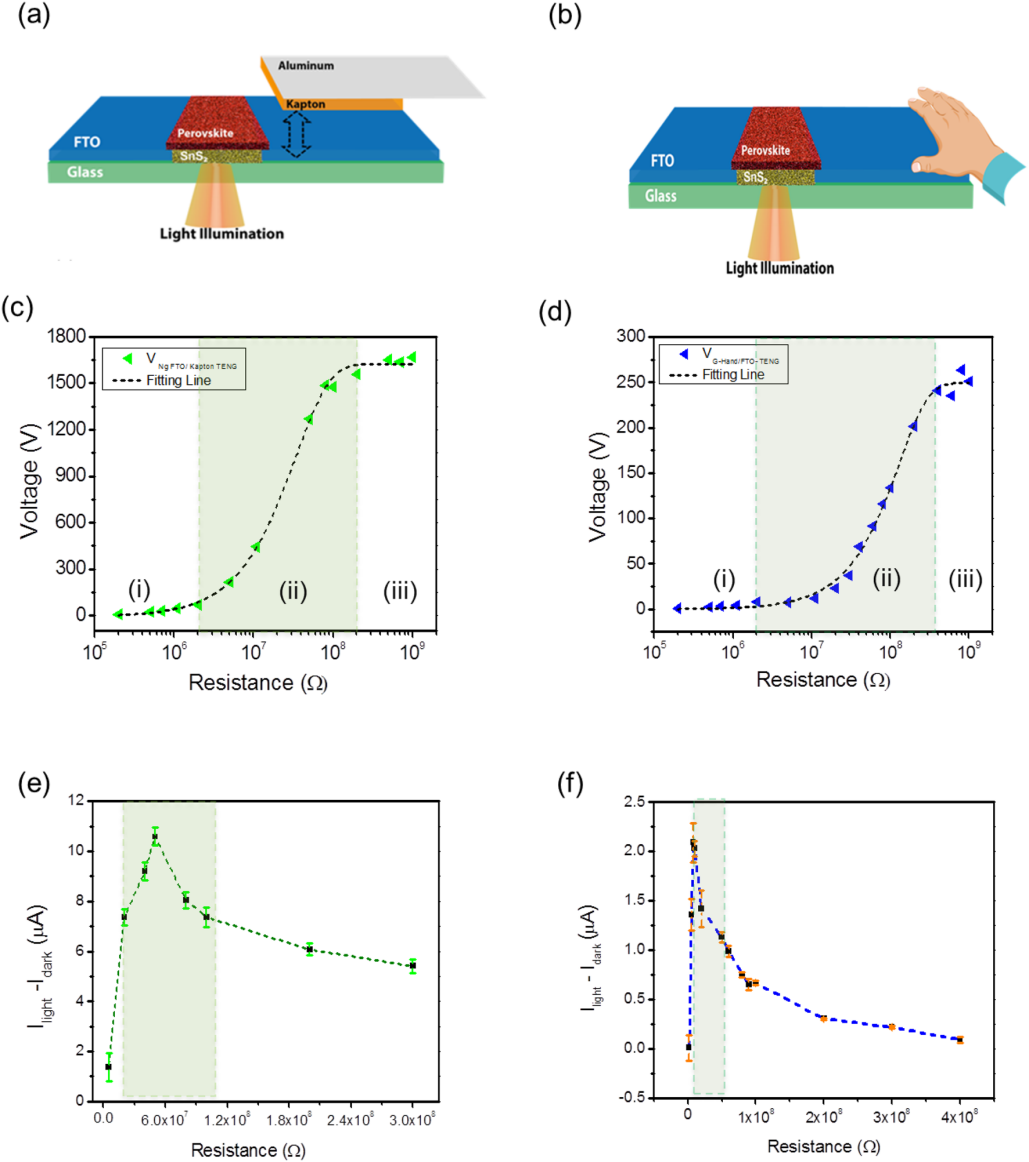
The Kapton/FTO and hand/FTO based TENG properties. Schematic representation of the self- powered SnS_2_/PVK based on (**a**) Kapton/FTO and (**b**) hand/FTO TENGs. The evolution of the output voltage vs. different load resistances for (**c**) Kapton/FTO and (**b**) hand/FTO TENG. The variation of *I*_*light*_*–I*_*dark*_ vs. load resistance for (**e**) Kapton/FTO and (**f**) hand/FTO TENGs.

To obtain the integrated self-powered photodetector, the SnS_2_/PVK PD was driven by tapping the Kapton and hand on the device’s substrate, which is FTO, as shown schematically in Fig. 4a,b, respectively. A white LED with different intensities of output light was used to illuminate the back-side of SnS_2_/PVK PD to achieve the ambient conditions for self-powered integrated PD circuits.

The V_OC-Ng_ of FTO/Kapton TENG has the highest amount of 740 V by applying the frequency and the applied for contacting and separating modes force is 2.5 Hz and 6 N, respectively (Fig. S6a). As the non-grounded configuration of single electrode (SE) is meaningless, the grounded circuit with one voltammeter is used for measuring the V_OC_ of SE hand/FTO TENG as shown in Fig. S6b. the average Voc of hand/FTO TENG is about 1.5 V. Figure S6c,d show the current and power density evolution of the FTO/Kapton and hand/FTO TENG versus the load resistance. It’s clear that, the highest amount of current and power density for the first, are 46 µA and 360 µW/cm^2^, respectively while these parameters are reduced significantly to about 5.2 µA and 2.8 µW/cm^2^ for the hand/ FTO TENG. The resistance zone of high power density for these TENGs are 11 MΩ < R _PMax- FTO/Kapton_ < 100 MΩ and 10 MΩ < R _PMax- hand/FTO_ < 500 MΩ, as shown in highlighted part of Fig. S6c,d, respectively.

As expected, the non-grounded output voltage of the CS-FTO/Kapton (Fig. 4c), shows three regions of low (i), medium (ii) and large (iii), and the active region ii is in the range of 20 MΩ < R_Ng-V-Kapton/FTO_ < 200 MΩ (the highlighted part in Fig. 4c). Similarly, Fig. 4d displays these three regions for FTO/hand TENG out-put voltage versus different load resistances in series. The critical zone of this TENG is located in the range of 10 MΩ < R _V-FTO/hand_ < 380 MΩ (highlighted part of Fig. 4d). Consequently, the common resistance limitation of the power supply density and the output voltage active zone for FTO/Kapton and FTO/hand are 20 MΩ < R_COMN-Kapton/FTO_ < 100 MΩ and 15 MΩ < R_COMN-hand/FTO_ < 380 MΩ, respectively.

The effect of R_L_ on (*I*_*light*_*–I*_*dark*_*)* is examined and the range of resistance is about 20 MΩ < R_ΔI-FTO/Kapton_ < 100 MΩ and 45 MΩ < R_ΔI-hand/FTO_ < 200 MΩ for the self-powered PD based on FTO/Kapton and FTO/hand as shown in Fig. 4e,f, respectively. As predicted, these restriction resistances (the highlighted part of Fig. 4e,f) are subsets of R_COMN-Kapton/FTO_ and R_COMN-hand/FTO_, respectively.

In Table 1, all resistance parameters summarized and their effective range on performance of the SnS_2_/PVK photodetector coupled with the three different GO paper/Kapton, FTO/Kapton and hand/ FTO TENG has been revealed.

**Table 1:** The summary of the resistance parameter range discussed in this study.

TENG	R_Pmax_ (MΩ)	R_Voutput-active Region_ (MΩ)	R_COMN_ (MΩ)	R_ΔI max_ (MΩ)
GO/Kapton	10–160	20–200	20–160	30–150
FTO/Kapton	11–100	20–200	20–100	20–100
Hand/FTO	10–500	10–380	10–380	10–60

R_PMAX_ = the resistance range in which the power density of the TENG has the highest amount (2/3 of the maximum amount of the power density).

R _Voutput- region II_ = the resistance range which leads to the appearance of the active zone in the output voltage (region ii).

R_COMN_ = the common resistance of R_PMAX_ and R_Voutput –region II_.

R_ΔI max_ = the resistance range which provides the most difference between the amplitudes of current under illumination and in the dark.

In order to compare the performance of these three TENGs as a power supply for SnS_2_/PVK PD, the load resistance equal to 50 MΩ which is in the range of R_ΔI-max_ for all TENGs, has been selected.

**Figure 5:**
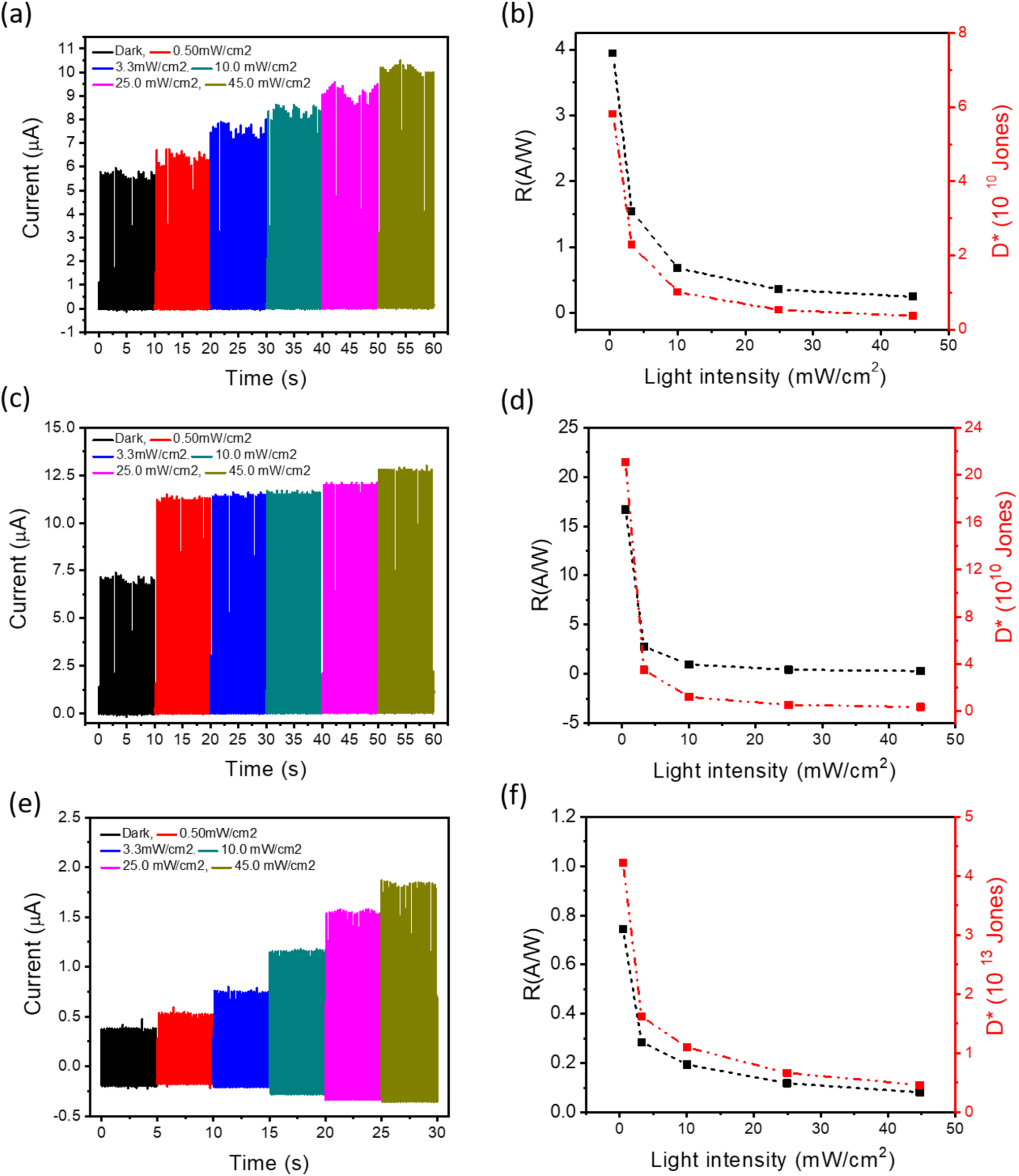
The self-powered SnS_2_/PVK PD supplied by different TENGs. The current amplitude of the self-powered SnS_2_/PVK supplied by (**a**) GO/Kapton (**b**) FTO/Kapton (**c**) hand/FTO. The R and D* parameters of the self-powered SnS_2_/PVK supplied by (**b**) GO paper/Kapton (**d**) FTO/Kapton (**e**) hand/FTO under different light intensities of illumination by white LED.

The diversity of current amplitude under different intensity of white light for self-powered PD supplied by the GO paper/Kapton, FTO/Kapton and FTO/hand is displayed Fig. 5a,c,e, respectively. The illumination power dependence of photocurrent is described with I_PC_ ~ P^α^, which is power-law relationship. For an ideal p-n junction based photodetector, it is expected that photocurrent depends linearly on the illumination power density, namely α = 1. However, because the introduced mid-gap states related to disorder, defects, or impurities may assist as traps or recombination centers, a sublinear (0 < α < 1) power dependence is ubiquitous. The dependency of current to incident light and the calculated α parameter by fitting the curve for the self-powered SnS_2_/PVK based on GO paper/Kapton, FTO/Kapton and hand/FTO TENGs has been illustrated in Fig. S7a–c, respectively. The average α parameter for this self-powered photodetector which is about the 0.77 is very similar to amount of this parameter when this photodetector has been supplied by the DC voltage as reported before^32^. Figure 5b,d,f display the evolution of the responsivity and detectivity parameters of proposed self-powered photodetector which tends to decrease at high light intensity. This may be ascribed to the increased carrier recombination with increasing light intensity^56^. Certainly, under low-intensity illumination, the electrons and holes that separated from photo-generated electron–hole pairs migrated to the SnS_2_ and perovskite surface, respectively and occupied their surface states. As the intensity of the illumination light was increased, more electron–hole pairs were generated, until all the surface states were filled at a certain intensity. When the intensity was enhanced further, the additional electron–hole pairs would recombine instantaneously as soon as they were generated (on the order of several tens of picoseconds). Hence, these charge carriers do not affect the charge transfer process^57^. This is a reason why R became nearly saturated at high light intensity. This trends have been seen and described in the other papers as well^58^.

The calculated normalized current amplitude values and detectivity of self-powered SnS_2_/PVK photodetector under the illumination of white light with 10 mW/cm^2^ are summarized in the Table 2.

**Table 2:** Comparison table of the PD’s parameters calculated for the self-powered SnS_2_/PVK supplied by different TENG.

TENG structure	R_L_ = 50 MΩ	$\Delta I/{I}_{0}$	D*(10^10^ Jones)
GO paper/Kapton		1.10	2.56
FTO/Kapton		1.21	2.83
FTO/hand		2.02	110

Take a closer look of the performance of the self-powered SnS_2_/PVK photodetector supplied by three different TENG with regarding the proper load resistance of the 50 MΩ, shows that the $\Delta I /{I}_{0}$ of the self-powered photodetector for integrated system, powered by hand tapping on FTO, is more significant than the other two TENGs as power suppliers. Interestingly, the magnitude of the D* parameter in the FTO/hand circuit is more than one hundred times greater than those of the GO paper/Kapton systems. In fact, as this configuration can detect the lower dark current, higher amount of detectivity parameter for photodetector is more probable. To examine the effect of hand tapping intensity and to prevent human error, the self-powered integrated SnS_2_/PVK supplied by touching the FTO with fingers, has been investigated. The current amplitude and the calculated R and D* vs. light illumination intensity, has been shown in Fig. S8a,b, respectively. In this situation the R and D* are decreased gradually with the light intensity and the D* of the device under illumination of white light with 10mW/cm^2^ is 6.15 × 10^12^ Jones, which is more than GO-paper/Kapton and FTO/Kapton based system. In the literature, the self-powered photodetector based on CH_3_NH_3_PbI_3_ perovskite with the nanorods and nano/microwires morphology with the complicated planar structure through high cost heterojunction metal deposition shows the highest amount of the responsivity and detectivity about 2.2 mA/W and 1.76 × 10^11^ Jones for nanorods^59^ and 161 mA/W and 1.3 × 10^12^ Jones for micro/nanowires morphology^60^, respectively. Moreover, the report on the self-powered PdSe_2_/ Perovskite fabrication shows the calculated R and D* for this device were about 313 mA W^−1^ and 2.72 × 10^13^ Jones by adopting lower light intensity of 35.1 μW cm^−2^ at 0 V^61^. So, the responsivity and detectivity of the proposed self-powered SnS_2_/perovskite described in this study in compare to the other planar self-powered photodetector based on the TENG is admirable.

**Figure 6:**
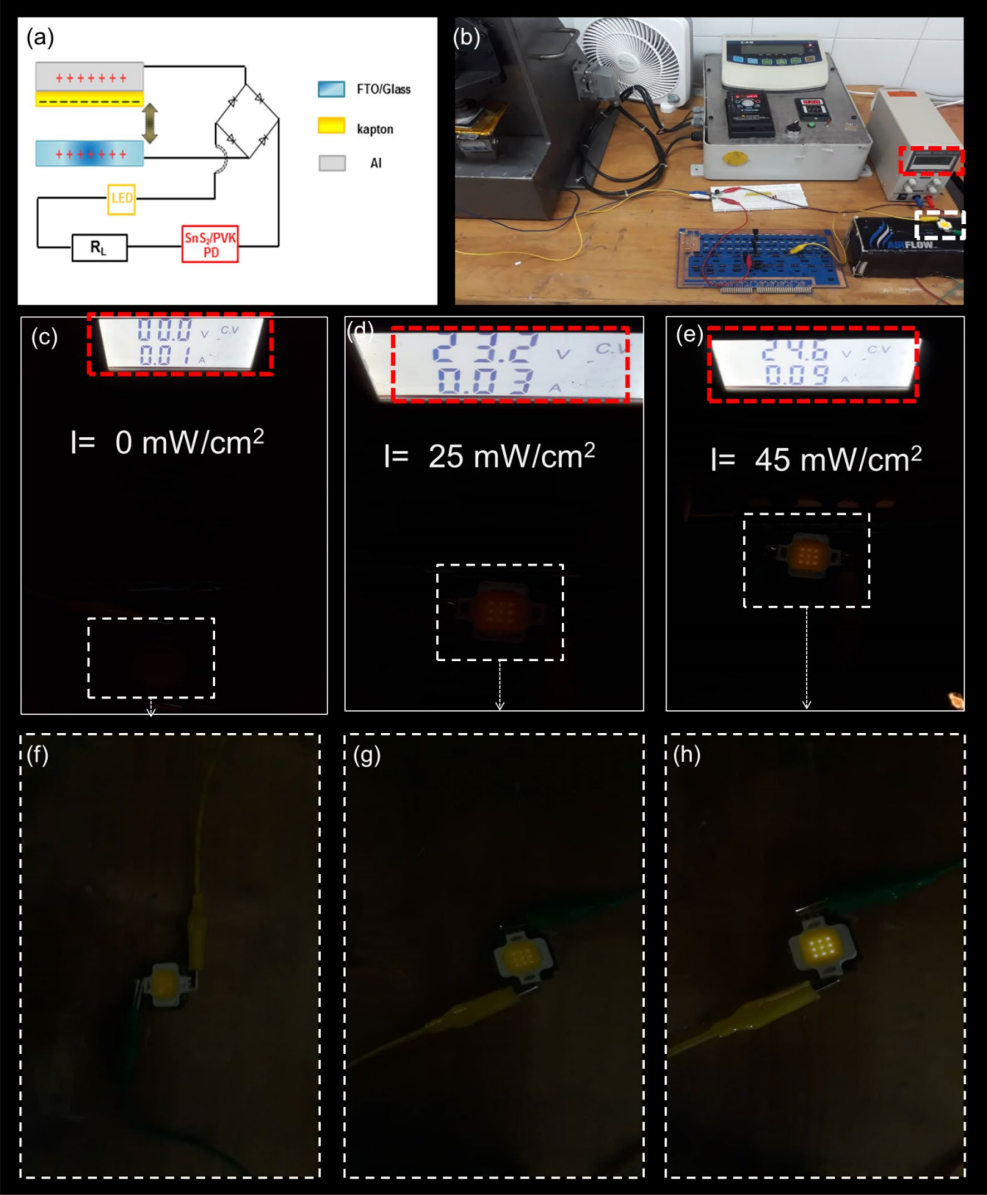
The performance of the integrated SnS2/PVK self-powered PD by using the LED marker display. (**a**) schematic and (**b**) the photograph of the photoelectric detection of the self-powered SnS_2_/PVK PD by FTO/Kapton by using rectifier bridge, load resistance and LED as the marker of detecting the white light illumination with different intensities. The brightness of the LED marker in the circuit under (**c**) and (**f**) dark conditions and white light with (**d**) and (**g**) 25 mW/cm^2^ and (**e**) and (**h**) 45 mW/cm^2^ intenisites.

At the end, the self-powered photodetection system was adjusted by the proposed circuit in this study as shown Fig. 6a. Although the other study used several LED in series as the marker for light detection, in this research, the FTO/Kapton TENG was connected to the rectifier bridge which is in series with the load resistance of 50 MΩ and one 10 W white LED (Fig. 6b). This simple circuit has been utilized just to show the potential of this study for optimizing the self-powered PD’s performance. As the DC power supply provides no voltage, which means no illumination on the SnS_2_/PVK PD, the LED marker displays a very low brightness (Fig. 6c,f), by increasing the intensity of the illuminated light to 25 mW/cm^2^ and 45 mW/cm^2^, the LED marker’s brightness increases remarkably which shows the decrease of the PD’s resistance in the circuit as it is shown in Fig. 6d,g,e,h, respectively. Of course this circuit comprises the load resistance to have effective performance of the self-powered SnS_2_/PVK.

So through designing of integrated self-powered PD, the wasted mechanical energies can be reused to detect even weak signals of the ambient light. Subsequently, using this integrated self-powered system in the proper design of circuits for some bio-medical applications such as heart rate monitoring^62^, which needs to detect the wavelengths in the visible range, could be more practical.

## Conclusion

In this research, a self-powered SnS_2_/PVK photodetector based on GO/Kapton TENG was developed through impedance matching circuit. For investigation of the effect of load resistance on the current circuit model, the proposed planar SnS2/PVK photodetector with no need to high cost and vacuum processes and the proper dark resistance was studied achieve the highest performance of the device. In this study, it was determined that the amount of load resistance should be in the common resistance range which generates the high power density as well as the active zone of the output voltage of the TENG. For the GO paper/Kapton TENG this common resistance range is 20–160 MΩ and the resistance range in which the *I*_*light*_*–I*_*dark*_ detected by SnS_2_/PVK PD becomes upper, is 30–150 MΩ, which is placed in the R_COMN_ variations. In the latter stage, integrated self-power SnS_2_/PVK photodetection system was investigated by tapping on the FTO by Kapton and hand separately. It is understood that by designing the integrated self-powered SnS_2_/PVK supplied by tapping the hand on the substrate, the amount of the dark current can be decreased which leads to enhance the detectivity parameter of the integrated self-powered SnS_2_/PVK This study describes the importance of designing of the self-powered photodetector for better performance which is the pave a way for designing the efficient self-powered sensors based on the TENG through impedance matching circuit.

## Experiments

### Material and characterization

The FTO/glass substrates (TCO30-10, 10 Ω/sq) were obtained from Sharif Solar Co. The organic compound, methylammonium bromide (MABr) and Foramidinium iodide (FAI), Cesium iodide (CsI) were purchased from Sigma Aldrich, and the other solvents were acquired from the Merck Company.

X-ray diffraction (X’ Pert Pro, PANalytical), local optical microscopy and field emission scanning electron microscope (FESEM, TESCAN, MIRA3) were used to study the crystallization, morphology, and structural characteristics of the samples.

### Photodetector’s preparation

The SnS_2_/ PVK photodetector fabrication was performed through CVD followed by spin coating process as reported recently^63^. Briefly, the 25 µm strip has been physically etched on the FTO substrates using laser Nd:YAG laser, QCW fiber, wavelength of 1064 nm and 10 Watt laser cutter (Connect laser technology Co. LTD). Then the laser patterned FTO was first cleaned sequentially with acetone, ethanol, and DI water in ultrasonic bath for 10, 5 and 5 min respectively. In this regard, a ~ 25 µm gap on the FTO substrate created by physical etching using laser Nd:YAG laser, QCW fiber, wavelength of 1064 nm and 10 Watt laser cutter (Connect laser technology Co. LTD).

To achieve vertically aligned SnS_2_ nanosheets grown on the FTO substrate through modified CVD process, 500 mg of elemental sulfur has been used and heating treatment was performed at 500 °C for 1 h, while they were 20 cm distant from each other in quartz tube. (Fig. S9a–c) The perovskite precursor solution was prepared by mixing PbI_2_, PbBr_2_, FAI, and MABr in anhydrous DMF:DMSO (4:1). Separately, the CsI standard solution in DMSO solvent was stirred overnight at room temperature and then added into the precursor solution with an equal volume percentage. The final precursor solution of triple cation lead perovskite, Cs_0.05_(FA_0.83_ MA_0.17_)_0.95_Pb(I_0.83_ Br_0.17_)_3_ was stirred overnight. The PVK layer was spin-coated via two steps of 1000 rpm and 4000 rpm for 10 s and 30 s, respectively (Fig. S9d); and chlorobenzene was utilized as the anti-solvent at the final 5 s of the second step to complete the crystallization of the PVK layer. Then the structure was baked at 100 °C for 1 h (Fig. S9e). The prepared photodetector was analyzed with the light illuminated from backside of the substrate (Fig. S9f).

The optoelectronic measurement of the device was carried out using KEITHLEY 6487 picoamperometer voltage source instrument. Light sources were arranged through several LEDs with 625 nm, 520 nm, 445 nm, 415 nm, and 395 nm wavelengths.

To measure the rising and falling time of the photodetector, an operational amplifier circuit and an oscilloscope have been used and the illumination with 445 nm and 5 mW/cm^2^ has been used.

To fabricate the all-oxide FTO/ZnO/Cu_2_O/Au photodetector, ZnO and Cu_2_O layers were deposited on FTO through electrodeposition method by using 0.08 M of Zn(CH_3_COO_2_).2H_2_O and 0.2 M of Cu_2_SO_4_ in a mixture with 0.3 M lactic acid, respectively. Both depositions were performed in a three electrode system with Ag/AgCl (0.1 M KCl), FTO and Pt as reference, working, and counter electrodes, respectively. The applied voltage for ZnO and Cu_2_O layer deposition was about − 0.85 V and − 0.65 V, respectively and deposition durations were about 20 min and 60 min, respectively. Then Au contact was deposited by thermal evaporation method as the top electrical contact. White light was illuminated from the back side of the glass/FTO substrate to measure the photocurrent behavior of the device. Optoelectronic measurements of the vertical all oxide structure of FTO/ZnO/Cu_2_O/Au were carried out by potentiostatic-galvanostatic system (µAuto-lab system, Metrohm)

### Triboelectric nanogenerator preparation

In order to prepare the self-powered system, CS-TENG was used to power the SnS_2_/PVK photodetector. The first CS–TENG was based on GO paper and Kapton layer. The GO was synthesized from natural graphite powder through modified Hummers method. In the next step, the GO paper was prepared by molding the concentrated GO suspension (~ 7 mg/mL) followed by drying in ambient conditions. The electrode was prepared by transferring the GO paper on a soft supporting layer and using Al foil as the back contact. The other electrode was prepared by covering the Al foil/ Kapton layer on the acrylic sheet as the supported layer. More in depth details about the applied TENG has been reported recently^42^. The second CS-TENG in this research was based on the FTO/glass and Kapton layer; the back contact of the Kapton was Al foil, while the connection of the FTO/glass electrode was prepared by itself. The CS motion of two electrodes was tuned by gauges of a tapping device with 2.5 Hz frequency, the applied force is about 6 N. The GO paper/Kapton and FTO/Kapton CS-TENG had an effective dimension of 8 × 8 cm^2^ and 8 × 10 cm^2^, respectively. The maximum spacing distance of the electrodes was 2 cm. The third TENG was prepared by tapping the hand on the FTO and could be considered as a kind of single electrode mode of the TENGs. Since the alternating current (AC) signal output voltage of CS-TENG is not suitable to drive the photodetectors directly^52^, a rectifier bridge was used to convert AC signal to a direct current (DC). The voltage of the applied TENGs and the PDs were measured by DSO1022A digital oscilloscope (Agilent Technologies). Moreover, as the output current of the CS-TENG was relatively low and approximately at µA magnitude^64^, a potentiostatic-galvanostatic system (µAuto-lab system, Metrohm) was used to measure the current in the designed circuit.

## Supplementary Information

Supplementary Information.

## Data Availability

Derived data supporting the findings of this study are available from the corresponding author on request.
