# Supplementary material for: Design of effective self-powered SnS2/halide perovskite photo-detection system based on triboelectric nanogenerator by regarding circuit impedance
Source: Sci Rep. 2022 May 4;12:7227. doi: 10.1038/s41598-022-11327-0 (PMC9068926; doi:10.1038/s41598-022-11327-0)
Supplement: Supplementary file 1 — Supplementary Information. [file 41598_2022_11327_MOESM1_ESM.docx]

# Design of effective self-powered SnS_2_/halide perovskite photo-detection system based on triboelectric nanogenerator by regarding circuit impedance

Leyla Shooshtari^a^, Soheil Ghods^b^, Raheleh Mohammadpour^a,*^, Ali Esfandiar^b^, Azam Iraji zad^a,b^

^a^-Institute for Nanoscience and Nanotechnology, Sharif University of Technology, Tehran, 14588-89694, Iran.

^b^-Physics department, Sharif University of Technology, Tehran, 11365-9161, Iran.

Corresponding author:

Institute for Nanoscience and Nanotechnology, Sharif University of Technology, Tehran, 14588-89694, Iran.

Tel.: +98 216616 4121; Fax: +98 2166164119.

E-mail: [Mohammadpour@sharif.edu](mailto:Mohammadpour@sharif.edu)

***Supporting Information***

Fig. S1-(a) Optical image of the SnS_2_ nanoflakes grown through CVD method. (b) FESEM image of top-view of vertical SnS_2_ nanosheet grown on the laser patterned FTO/glass as the substrate; this figure shows the growth of SnS_2_ nanosheets make a bridge crossing over the strip on the FTO substrate.

| 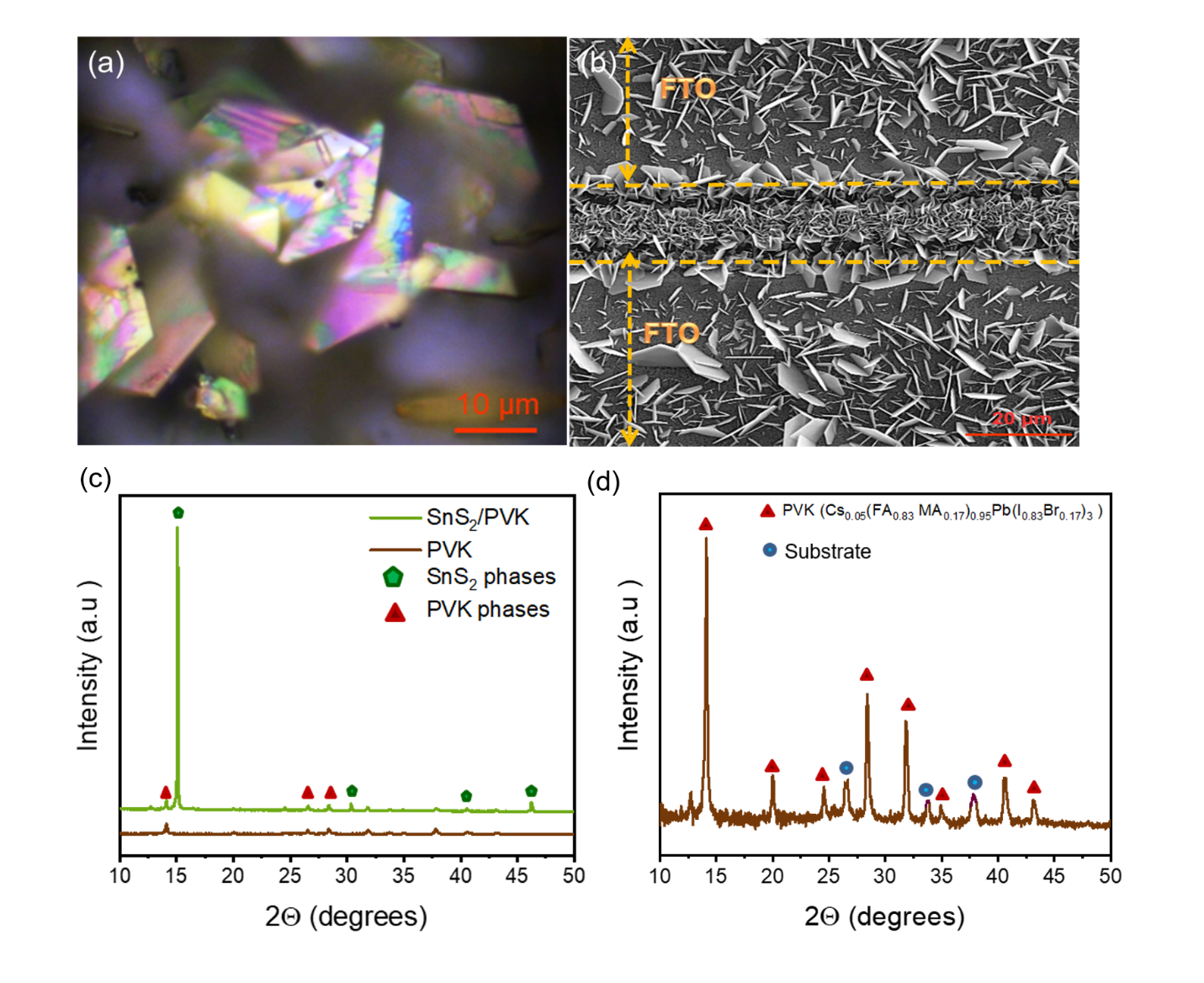 |
| --- |
| **Fig.S1 (a) Optical image of the SnS_2_ nanosheets grown through CVD method in this study. (b)The FESEM analysis of vertically grown SnS_2_ nanosheets on the patterned FTO laser. The XRD pattern of (c) SnS_2_/PVK and PVK layers together and (d) pure PVK lonly** |

Fig. S2- Characterization of SnS_2_/PVK photodetector

| 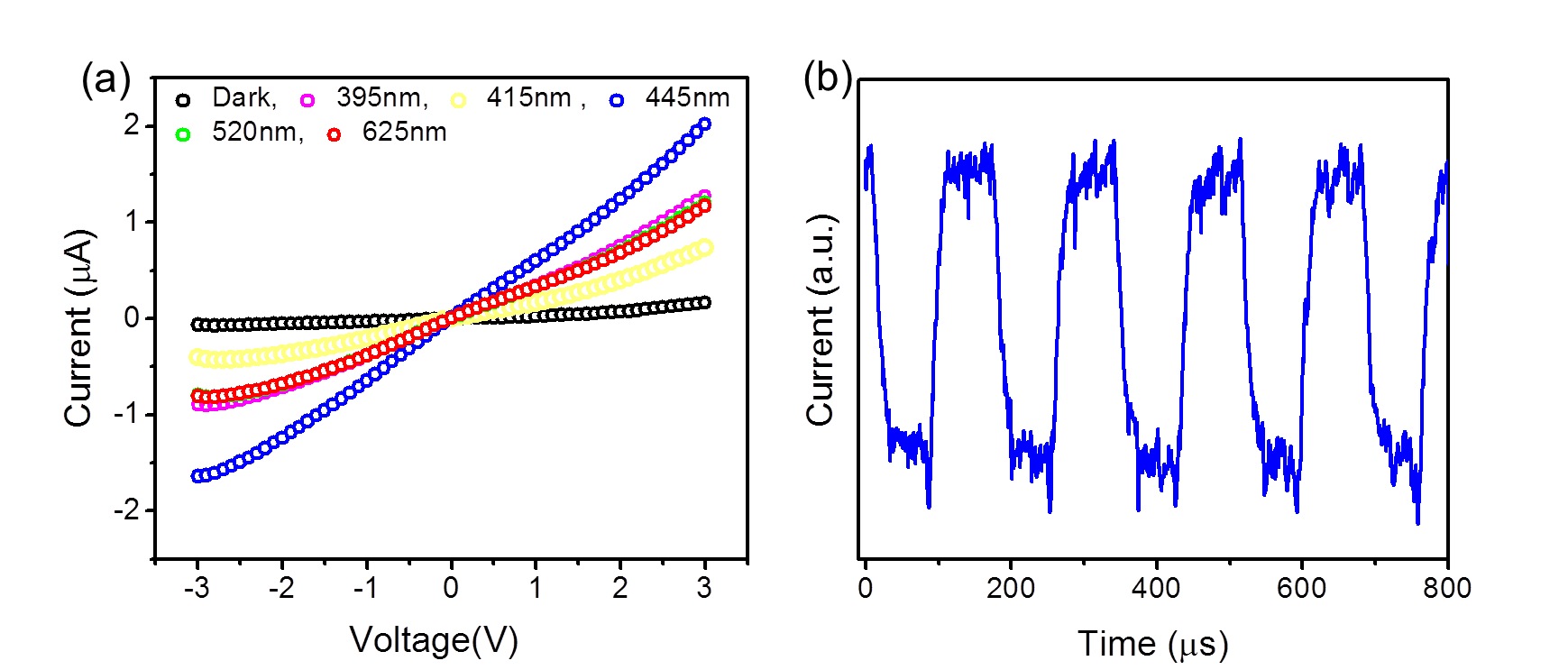 |
| --- |
| **Fig S2. (a) the *I-V* plot of the SnS_2_/PVK photodetector under illumination of different wavelengths at the same intensity of 5mW/cm^2^ (b) the evolution of the current of the SnS_2_/PVK vs. time under the wavelength of 445 nm** |

Fig S3. All oxide based PD characterization.

| 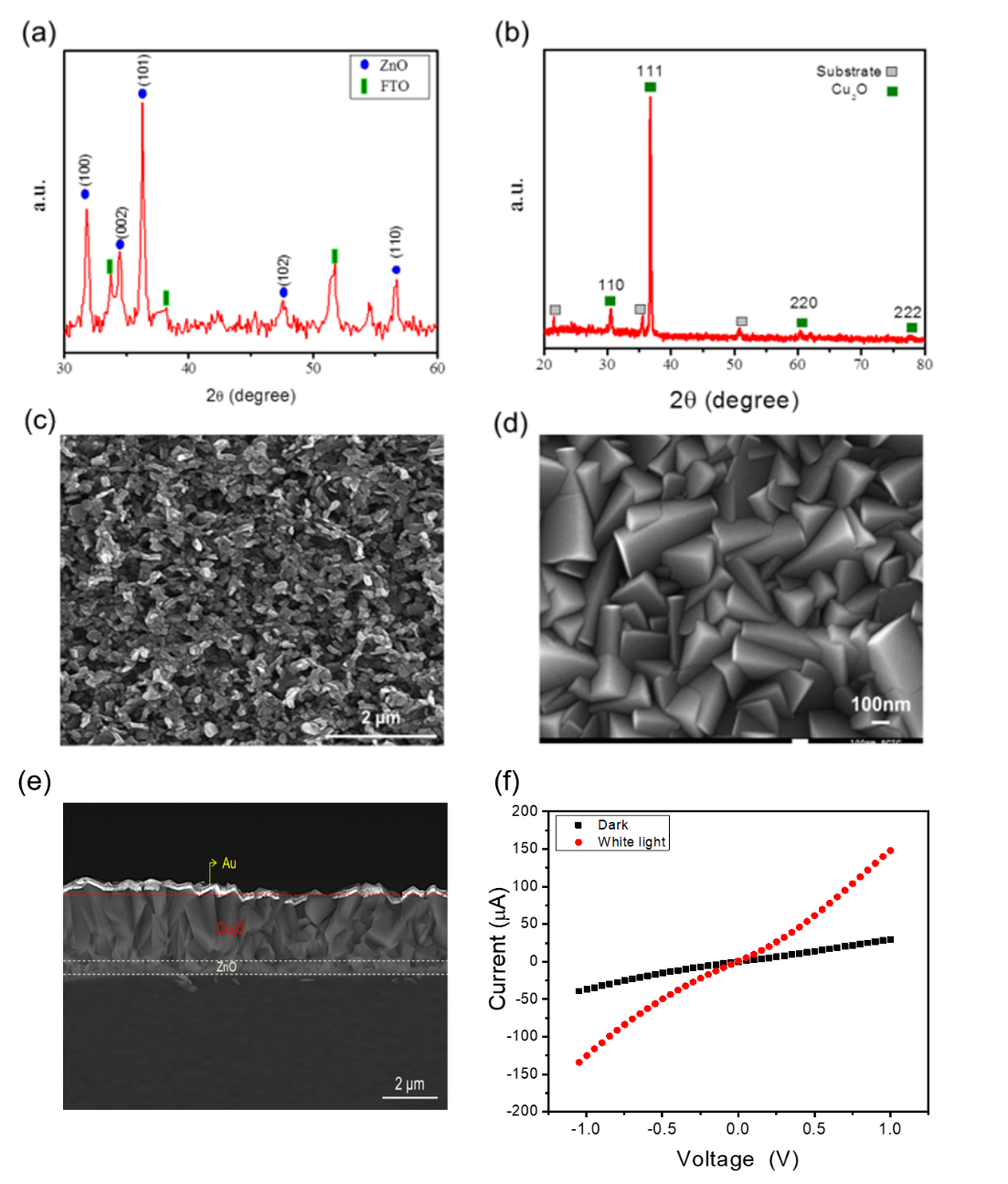 |
| --- |
| Fig S3. The XRD plot of the (a) ZnO and (b) Cu_2_O and top-view FESEM analysis of the (c) ZnO and (d) Cu_2_O electrodeposited layers on the transparent conductive oxide substrate. (e )The FESEM analysis of a cross-section of the FTO/ZnO/Cu_2_O/Au structure. (f) The *I-V* curves of the all oxide photodetector in the dark and under incident white light |

Fig S4: the schematic of the grounded and non-grounded models to characterize the V_OC_ and output voltage of the CS-TENGs in this research.

| 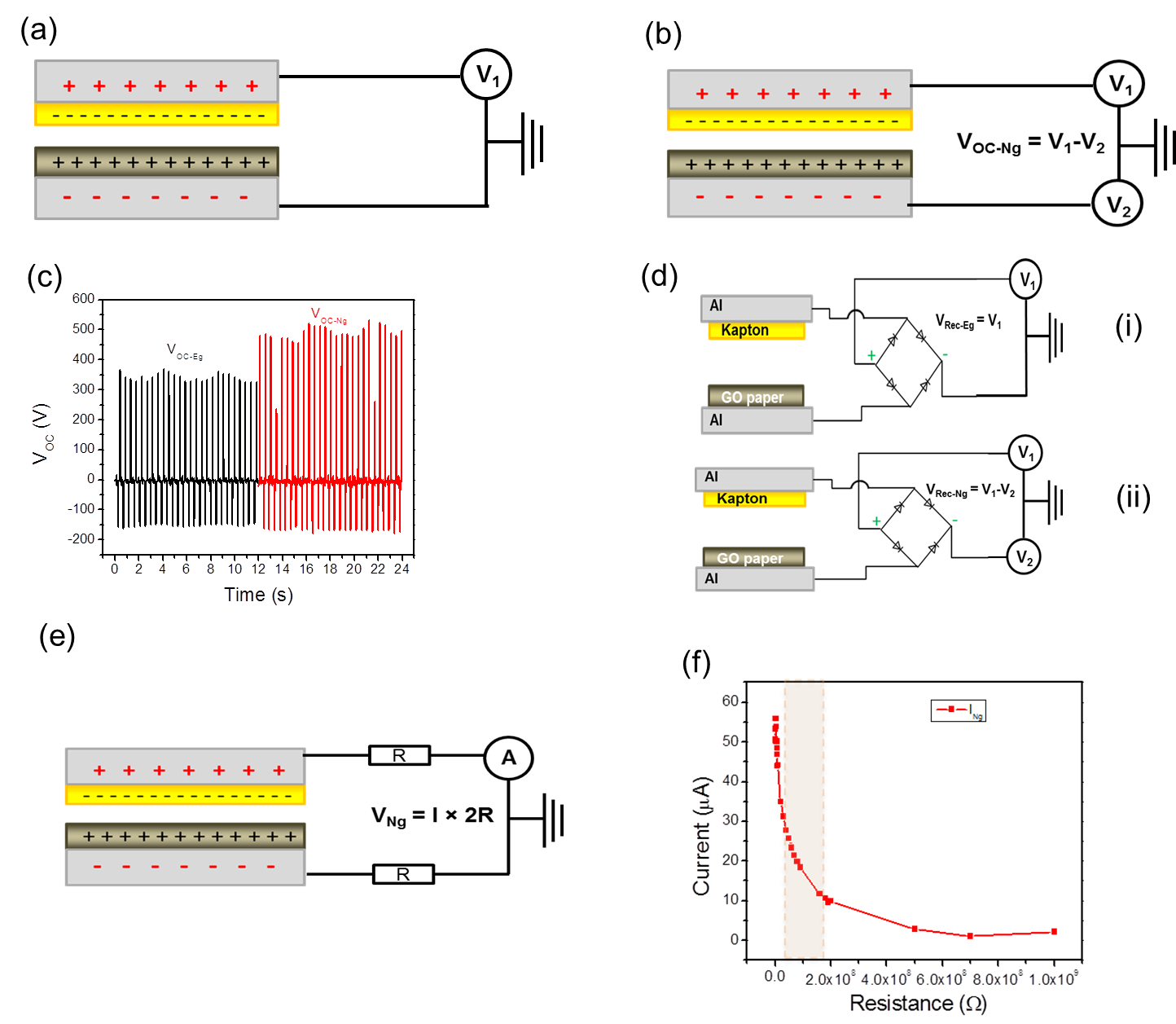 |
| --- |
| Fig S4. The schematic of (a) the grounded (Eg) and (b) non-grounded (Ng) systems to measure the V_OC_ of the CS-TENG. (c) the out-put voltage vs. time in the grounded and non-grounded configuration of the GO paper/kapton based TENG. The schematic of (d) the grounded (i) and non-grounded(ii) systems including the rectifier bridg to measure the V_OC_ of the TENG. (e) the schematic of the non-grounded model to measure the current and the out-put voltage of the TENG. (f) the current evolution of the GO paper/kapton based TENG vs. different load resistances in the circuit |

Fig S5: The current amplitude of the self-powered SnS_2_/PVK photodetection system based on GO/kapton TENG under different conditions

| 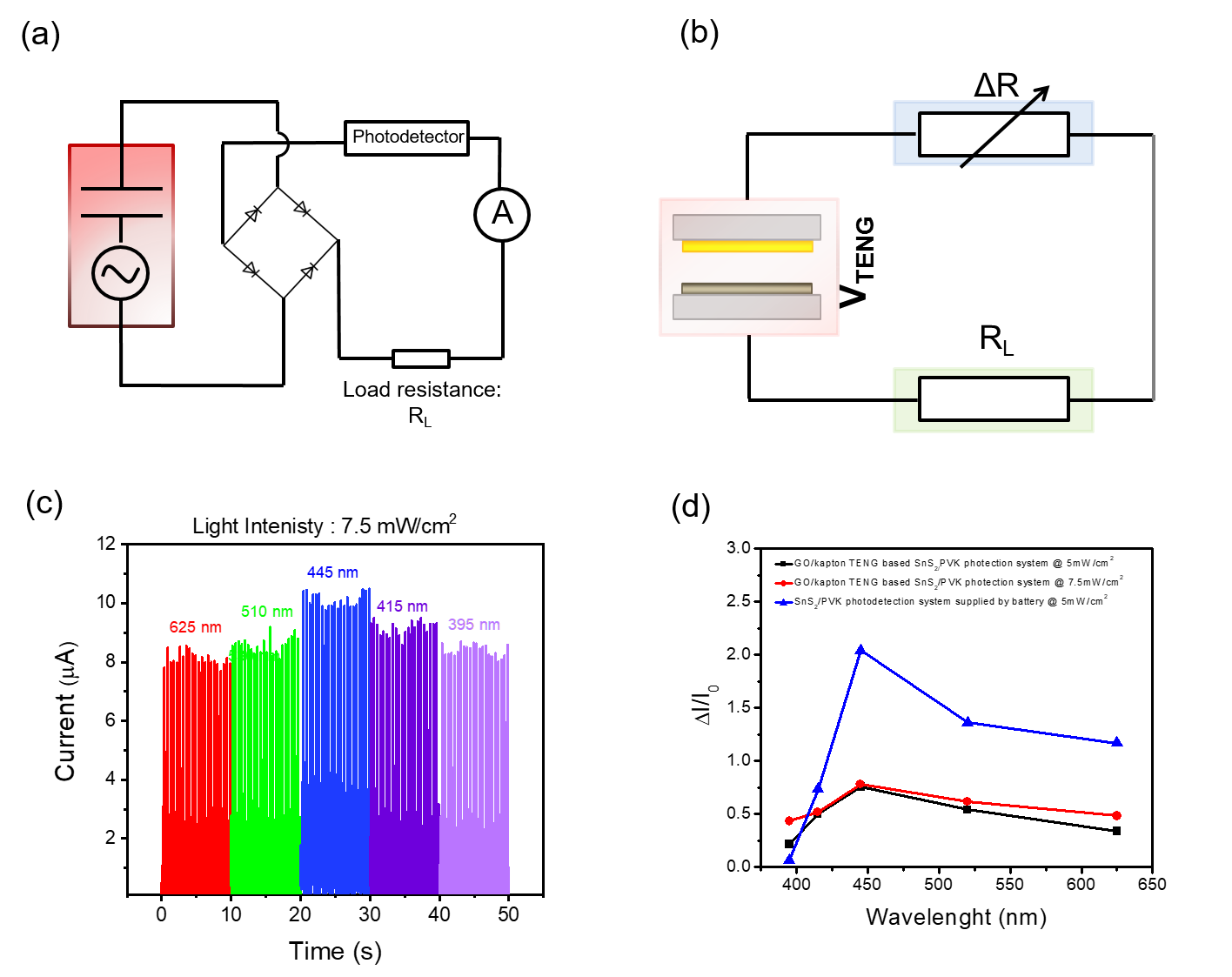 |
| --- |
| Fig S5. Schematic representation of (a) the current amplitude measuring circuit (b) the current circuit model used here to study the self-powered photodetection system powered by GO paper /kapton TENG. The evolution of (c) current amplitude and (d)$\frac{\Delta I}{I_{0}}$Vs.different incident of visible wavelength for different intensities of 5 mW/cm^2^ and 7.5 mW/cm^2^ for the self-powered SnS_2_/PVK photodetector supplied by GO/kapton TENG and for this photodetector which is supplied by a battery. |

Table S1:In this study, the dependency of self-powered PD’s resistance to the impedance of the TENG has been evaluated. As mentioned before the dark resistance of the FTO/ZnO/Cu_2_O/Au is in the order of 10 KΩ, and this resistance is out of the range of TENG’s resistance which produce great amount of the density power and the active zone of output voltage which is about MΩ. Even by inserting different amount of load resistances (2MΩ-100MΩ) in the self-powered impedance matching circuit TENG, intangible difference in current amplitude in the dark and under illumination has been observed (Table S1). So, the other key point which should be considered for effective performance of the self-powered sensors powered by TENG, is matching the inner resistance of the device and impedance of the TENG.

| Table S1. The amplitude current amount of FTO/ZnO/Cu_2_O/Au in the dark and light whiledifferent amount of load resistance is in sries with amperometer in the impedance matching circuit by using GO/kapton TENG | | |
| --- | --- | --- |
| Load resistance (MΩ) | I_Dark_(A) | I_Light_(A) |
| 2 | 3.1E-5 | 3.22E-5 |
| 4 | 3.26E-5 | 3.2E-5 |
| 8 | 3.32E-5 | 3.24E-5 |
| 20 | 2.53E-5 | 2.45E-5 |
| 50 | 1.54E-5 | 1.58E-5 |
| 80 | 1.24E-5 | 1.21E-5 |
| 100 | 1.08E-5 | 1.1E-5 |

Fig S6: the characterization of the CS Kapton/FTO and the SE FTO/hand TENG

| 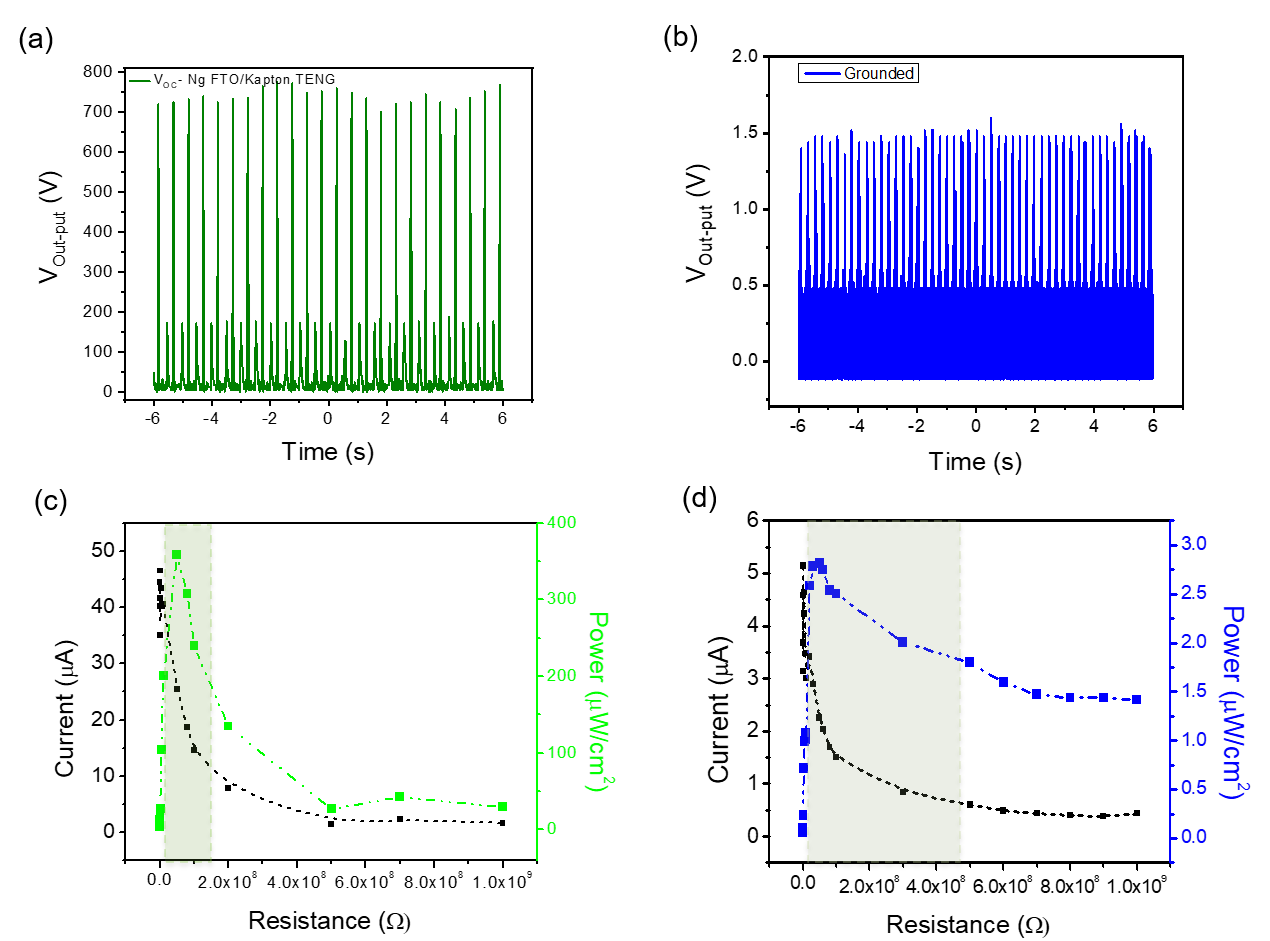 |
| --- |
| Figs S6. The out-put voltage of the (a) FTO/kapton in the non-grounded and (b) hand/FTO TENG in the grounded circuit . The evolution out-put current and power density of the (c) FTO/kapton and (d) hand/FTO TENG. |

Fig S7. The powere law calculation for the self-powered SnS2/PVK PD supplied by all TENGs

| 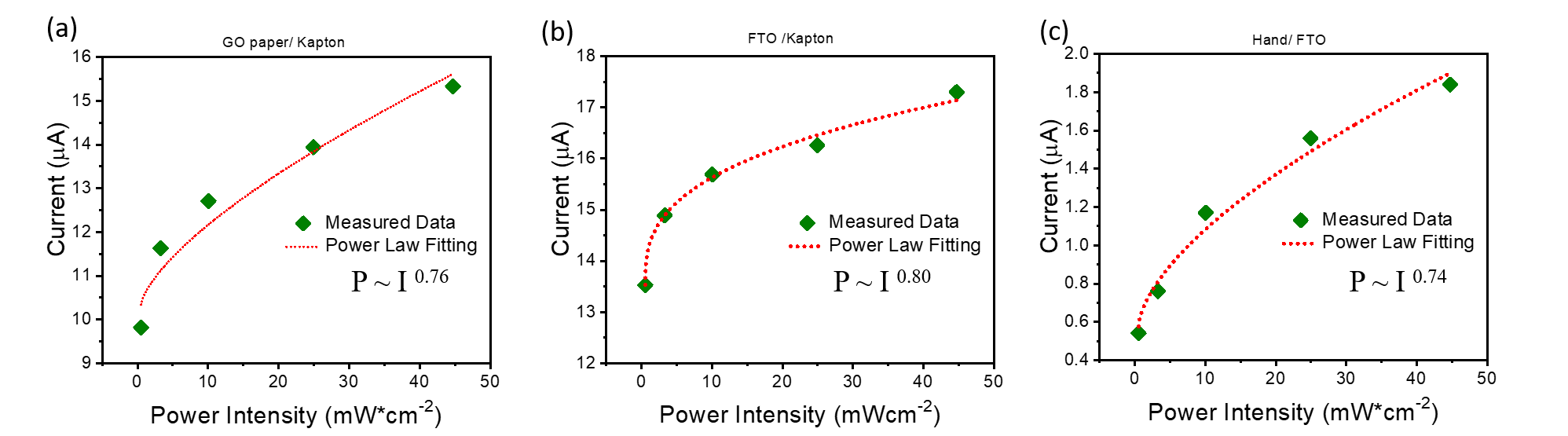 |
| --- |
| Fig S7. The photo-current and the power law calculation of the self-powered SnS2/PVK supplied by (a) GO paper/Kapton (b) FTO/Kapton and (c) FTO/Hand TENGs |

Fig S8. The self-powered SnS_2_/PVK PD coupled by touching the FTO with hand as the TENG

| 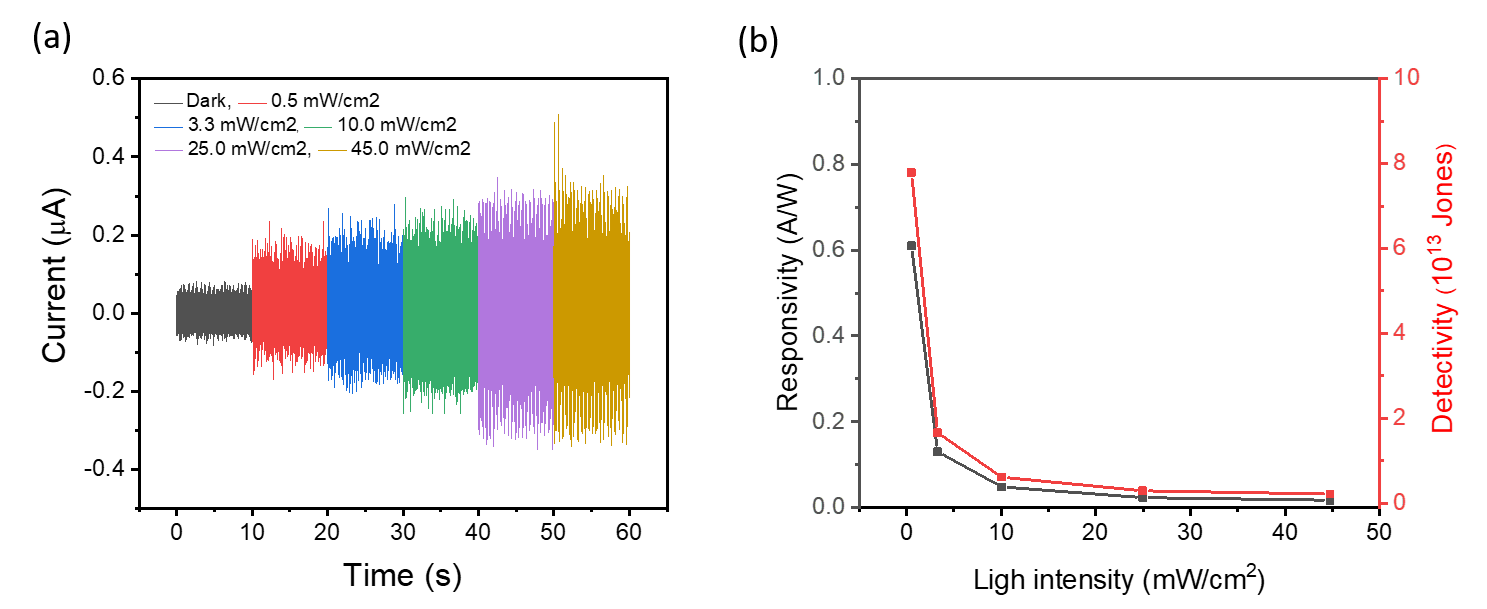 |
| --- |
| Fig S8: (a)The current amplitude and (b) the responsivity and detectivity of the self-powered SnS_2_/PVK supplied by touching the FTO with hand under different white light intensities |

Fig S9: fabrication of SnS_2_/PVK planar photodetector

| 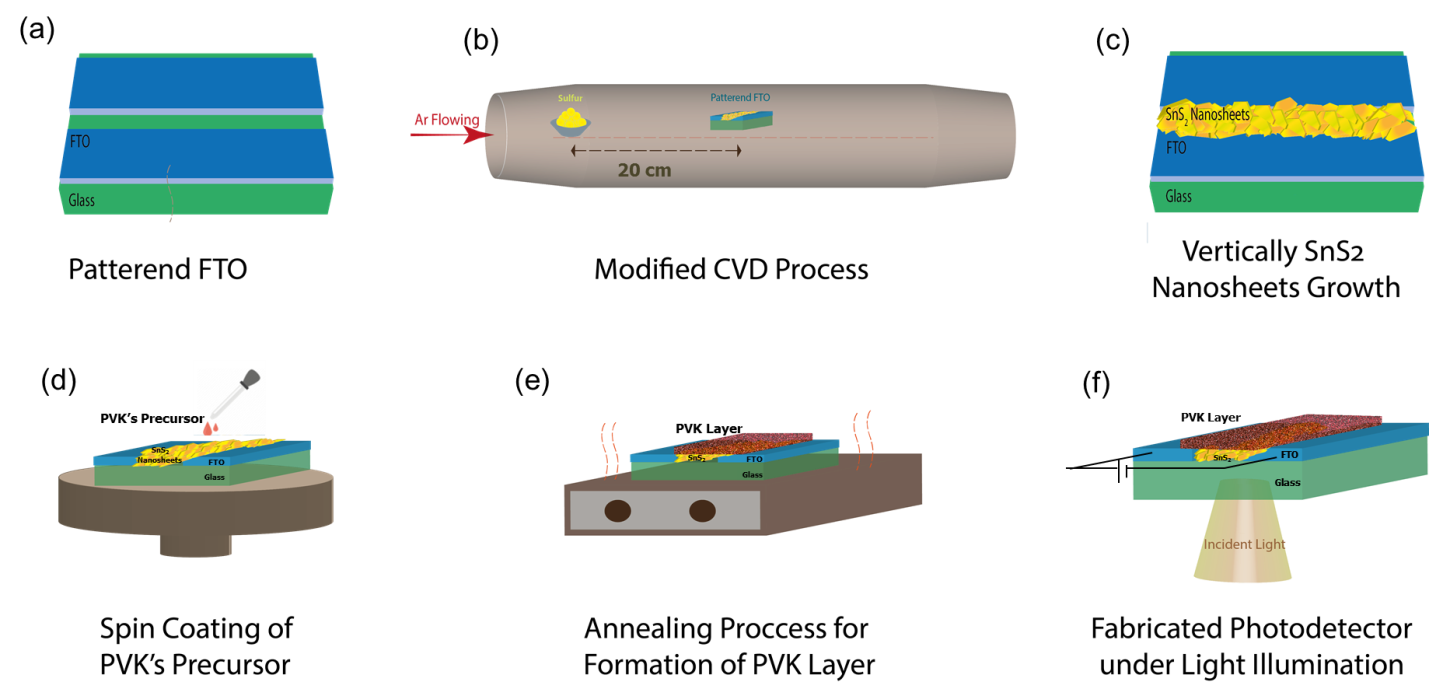 |
| --- |
| Fig S9. Schematic representation of the fabrication of SnS_2_/ PVK photodetector;  (a) laser patterned FTO, (b) Modified CVD process with applying 500 mg of sulfur source and using the FTO as both the inherent Sn source and the substrate, (c) the top view of vertical growth of SnS_2_ nanosheets which create a bridge between the FTO gap. (d) Spin-coating deposition of precursor solution of triple cation lead perovskite, Cs_0.05_(FA_0.83_MA_0.17_)_0.95_Pb(I_0.83_Br_0.17_)_3_ and the following anti solvent deposition process. (e) Annealing treatment at 100 ^o^C, as the final step of PVK depositions. (f) The optoelectronic measurement of the SnS_2_/ PVK photodetector, while specific wavelengths of the light are illuminated from backside. |
